# Predictors of medical cost and length of stay of motorcycle injury patients presenting to hospitals in Kisumu City, Kenya

**DOI:** 10.4314/ahs.v24i2.41

**Published:** 2024-06

**Authors:** Wilberforce Cholo, Wilson Odero, Japheths Ogendi

**Affiliations:** 1 Department of Public Health, Masinde Muliro University of Science and Technology; 2 School of Medicine, Maseno University; 3 Department of Public Health, Maseno University

**Keywords:** Motorcycle injuries, length of stay, cost, predictors, severity

## Abstract

**Background:**

Motorcycle crash injuries (MCIs) cause mortality and exert financial cost, globally. However, there is paucity of information on cost and length of stay of motorcycle injuries.

**Objective:**

To assess factors influencing medical costs and length of stay (LOS) of motorcycle crash injuries presenting in hospitals in Kisumu city,

**Methods:**

This was a cross-sectional study in which all 1073 motorcycle injury cases were enrolled over a period of six months. A pre-tested, questionnaire was used to collect data. Data were analysed using Analysis of Variance (ANOVA), logistic regression and multivariable regression analysis. P-value < 0.05 considered significant.

**Results:**

A total of 1073 motorcycle injury visits were made to the hospitals during the study period of which 46.0% were admitted. The total medical cost for motorcycle injury patients was Kshs. 19, 134,877 (USD 191348.77). The mean LOS was 19.8 days (1-235 days). Injuries requiring surgical intervention, higher injury severity score, and helmet non-use were significantly associated with higher medical cost and length of stay.

**Conclusion:**

Motorcycle injuries are a major cause of hospital use and present significant economic burden. Surgical intervention, injury severity and non-helmet use were the major predictors of length of stay and medical costs.

## Introduction

Road traffic injuries are predominant source of deaths, hospitalization, incapacity, and loss of income. Over 1.36 million road traffic fatalities and 20–50 million nonfatal injuries occur every year on global roads-1 Globally, motorcycle fatalities account for 28 %,^1^ and 7-16% in the WHO Africa region^1^.

Motorcycle crash injuries and deaths continue to rise globally^1^,^2^. This is due rapid motorization, poor enforcement of traffic safety regulations, and un- safe behaviors. The outcome of these injuries cause huge medical expenditure and intangible suffering^3^,^4^. Motorcycle crash casualties during a crash, experience and absorb a high energy impact since there is no external protection; consequently, sustain severe injuries to the head, extremities and spine resulting in longer hospital days and high cost^5^, ^6^ ^7^.

In the last decades medical cost has been rising and varies from country to country^8^. Studies in the high Income Countries (HICs) have documented motorcycle injuries to cost 12 B US$ annually^8^. Nine Billion in USA^9^ US$10,917 and US$ 16 767 037 in Argentina^10^ in the year 2010 and 2014 respectively. In LMICs the costs was US$ 63,128.30 in Ghana^11^, US$ 269,000 in Rwanda^12^; US$ 128169.3 in Nepal^13^ in 2011, 2015, and 2016 respectively.

Length of hospital stay (LOS) is an important measure to evaluate the burden of trauma care on hospitals and contributes to medical costs^14^,^15^. For instance, reduced hospital LOS has been associated with reduced costs^16^ while prolonged LOS is related to increased demand for resources both material and manpower^15^. Therefore, LOS is a useful indicator for costs, quality of care, and hospital resource utilization^17^. Previous studies from Brazil^18^, Jamaica^19^, China^7^, Taiwan^20^ reported hospital mean length of stay of 15.9 ± 23.8 days, 10±8 days, 10.6±11.5 days, 14±12.5 days respectively. Other studies have shown that length of stay is influenced by age, injury severity, gender, anatomic injury site, hospital mortality, insurance cover and hospital location^21^-^22^. One challenge in previous medical cost studies is that calculations of medical costs are limited to reviews of hospital charges at single centres^8^. Cost estimates derived from such reviews are incomplete, consequently under-costing the burden^23^ thus, true costs are under-represented.

In Kenya, in the year 2020, a total of 16970 people were injured and 3975 killed in road traffic crashes with an estimated cost of US$ 4 billion, of which 6143 and 1574 were motorcycle injuries and deaths, respectively^24^. Unfortunately, there is no data on the exact figure on the economic impact and the rate of admission of motorcycle related injuries. The number of motorcycle crashes in Kenya has increased from 141 in 2005, to 6143 in 2020 25,26. Previous studies in Kenya have indicated that the proportion of motorcycle injuries varies from 24%-62% of all road traffic injuries^27^,^28^,^29^. Other motorcycle related studies in the country have been done on safety practices and identified wearing helmets as an important behavioural measure in reducing motorcycle injuries and fatalities^30^,^31^. However, there is no study focused on hospital length of stay and medical costs incurred by motorcycle crash injuries and associated factors. Kisumu City has no reliable data on medical cost and hospital length of stay of motorcycle injury victims. The few available studies have been population based^32^,^33^ with no focus on length of stay and cost of care and associated factors. This is the first study to examine hospital length of stay and medical cost of motorcycle injuries in the Kenya. It is expected that data will assist hospitals to plan for care of motorcycle injury patients and policy makers inform focused interventions to reduce the burden of these injuries.

## Materials and methods

### Study Site and design

This study was carried out in Tier III Hospitals in Kisumu City located in western Kenya. Kisumu City has a population of 610,082 comprising of 52.3% aged 0-19 years, while 36.2% is between 15-45 years^26^. Fifty point two percent of the population are females while 49.8% are males. The main mode of transportation used within the Kisumu city is walking which comprise slightly more than one half (53%) of daily trips^34^. Motorcycle, matatus and bicycles comprise (19%), (13 %) and (4 %), of urban transport respectively^36^. Motorcycle traffic accident-related deaths and injuries present a major public health problem in Kisumu city^35^.

This was a descriptive prospective study of motorcycle crash injury patients of all age groups and gender that presented to the Emergency Departments (ED) of three Tier III hospitals in Kisumu City between May 2019 and November 2019. Tier III hospitals are County hospitals with comprehensive in patient, diagnostic, medical, surgical and rehabilitative care facilities, includes all Level 4 and Level 5 hospitals^36^. These hospitals were; Jaramogi Oginga Odinga Teaching & Referral Hospital Kisumu County Referral Hospital and Aga Khan Hospital^36^. Jaramogi Oginga Odinga Teaching and Referral Hospital (JOOTRH), Kisumu County Hospital are public hospitals run by the County Government of Kisumu^36^, while Aga Khan Hospital is a private hospital. These hospitals were deliberately selected because they all have well-equipped Emergency Departments that provide clinical care to the crash-involved patients on 24 hour basis.

### Sample size determination

All 1073 motorcycle injury patients who presented to the Jaramogi Oginga Odinga Referral Hospital (JOOTRH) and Kisumu County hospital (KCH), and Agakhan hospitals were consecutively enrolled for a period of six months. Using G. power analysis the sample size had sufficient effect size and power of 95% to detect meaningful differences at 5% level of significance using regression analysis. The distribution of number of motorcycle injury cases per hospital are as indicated in table 3.1.

**Table 3.1:** Distribution of injury cases

Hospital	For 6 months
Jaramogi Oginga Odinga Teaching and Referral Hospital	610
Kisumu County Hospital	310
Aga Khan Hospital	150
Total	1073

### Sampling Methods and Procedure

Total population sampling technique was used to recruit participants for the study. Total population sampling is a sampling technique in which every population element is recruited as samples in a study. The study enrolled all the patients presenting with motorcycle crash injuries in the Emergency Departments of the named hospitals for a period of six months. Several studies on length and costs have applied similar sampling approach^15^,^16^,^19^.

### Data collection

Data was obtained on all the motorcycle injury patients who presented to the Emergency Departments of the Tier III hospitals over a period of six months (from 6^th^ May, - 3^rd^ November, 2019). Patients with motorcycle injuries presenting at the hospitals' emergency department were screened 24-hours a day by research staff as close to time of arrival as possible during the enrolment period after the patients were examined by either a doctor or a clinical officer. The study was carried out from registration desks and examination rooms of the study hospitals. Patients that required admission were admitted to the admitting surgical firm. Injured patients gave their informed consent either after they were stabilized or relatives who brought motorcycle injury cases for care assented. All patients enrolled were initially assessed and cared for according to Advanced Trauma Life Support (ATLS) guidelines and contemporary standards of trauma care by attending physician in the Emergency department. Data were gathered on: demographics, road user category; anatomic injury site, helmet use; diagnostic procedures, treatment and patient disposition. The Length of hospital stay (LOS), calculated as the time interval between hospital admission and discharge and patient disposition were recorded from the inpatient notes. Casualties who were admitted and discharged on the same day were considered to have been hospitalized for 1 day. Data on anatomic site of injury and diagnosis were also collected: the pelvic girdle and the rest of the lower limb were categorized as lower extremities; the pectoral girdle constituted upper extremities; head injuries constituted injuries to the face, neck, and head including concussions; and thoracic injuries including rib fractures and visceralorgans within the thorax were considered as chest injuries.

Injury severity was determined using the Abbreviated Injury Scale (AIS), and Injury Severity Core (ISS) for each casualty. Severity assessment was done by the attending physician. The ISS measures the severity of the injury based on the Abbreviated Injury Scale (AIS), was computed by squaring the highest score in the three body regions most affected (head and neck, face, chest, abdomen, extremities, and external). The ISS score ranged from 0 to 75^39^, ^40^, and were categorized in four groups: ISS 1–8, 9–15, 16–24 and 24+. Further classification was done into minor and major injuries. Therefore, motorcycle injuries were classified into minor (ISS <16) and major injuries (ISS≥16)^41^ Glasgow Coma Scale (GCS) and vital signs at the time of arrival at the emergency department were taken. GCS score of below 8 was indicated as severe; 9-12 as moderate; and 13–15 as mild head injury.

Patients were followed up from the time of presentation to discharge from the hospital, referral or death. The diagnosis and clinical care of the patients, treatment outcomes, and, if deceased, cause of death were documented. Data were entered in pre designed structured questionnaire.

Medical costs, obtained from the hospital billing records, included the cost of transport to the hospital, costs of first aid services, consultation, laboratory tests, radiological tests, medicines, surgical procedures, cost of hospital bed per day, cost of food. These were summed up for each injury patient as the cost of treatment for the period of stay in the hospital accordingly. This data collection strategy has been used in previous studies^20^,^42^,^43^,^44^.

### Data analysis

The quantitative data were coded and entered into SPSS version 21 programme^45^.

Descriptive statistics was used to examine the frequency distribution of demographic characteristics of motorcycle injury cases, anatomic injury site and severity in relation to length of stay. The mean LOS, and medical cost were compared in sub-categories of motorcycle crash patients (age, sex, and injury characteristics) using analysis of Variance (ANOVA) and Chi- square. Log transformation of outcome variables was undertaken due to the skewed distribution of hospital charges and length of stay. Multivariable logistic regression analysis was used to identify the factors contributing the length of stay and medical cost. Standardization was done in the model to compute adjusted odds ratios. The analyzed data were summarized in tables upon which the references drawn. For all tests, the threshold of significance was fixed at 5% level with P-value less than 0.05 considered significant.

### Ethical considerations

Ethical approvals were obtained from Maseno University Ethics Review Committee (Ref. No. MSU/DRPI/MUERC/00649/18) and Jaramogi Oginga Odinga Teaching and Referral Hospital Ethical Review Committee (ERC.IB/VOL.1/578). Permission to collect data was obtained from each of the participating hospitals. All participants provided informed consent in either Kiswahili or English. All relevant information about the study were communicated to all respondents before the study was carried out. Patients with altered clinical instability who did not have sufficient awareness to make decisions were excluded given their inability to provide informed consent, however these patients were reassessed for enrollment if their clinical state allowed during their emergency department care course.

## Results

### Socio-Demographic Characteristics of motorcycle injuries

The demographic characteristics of the motorcycle injury patients are shown in Table 2. A total of 1073 motorcycle crash injury cases were recorded in the study hospitals during a 6-month period of which 494(46.0%) were admitted. Of the admitted motorcycle crash injury patients, over three quarters 383 (77.5%) were males, with male to female ratio of 3.5:1. The ages of the patients ranged from 2 to 84 years, with a mean of 29.6 (SD 12.2) years and median 31 years. The modal age group was in 21-30 years bracket. A greater portion of motorcycle injury patients completed secondary school 470(43.8%) of which 283 (51.9%) and 187 (37.8%) were treated and released at emergency department and admitted respectively.

**Table 2:** Demographic characteristics of motorcycle injury presentation to the hospitals

Characteristic of the motorcycle injured cases	Admitted to hospital		Treated and released in the ED	

	No.	%	No.	%
**Gender**				
Male	383	77.5	392	71.5
Female	111	22.6	156	28.5
Total	494	100	548	100
**Age**				
0-10	17	3.5	41	7.5
11-20	55	11.2	61	11.1
21-30	170	36.4	249	45.4
31-40	109	22.0	129	23.5
41-50	64	12.9	35	6.4
Above 50	79	16.1	33	6.0
Total	494	100	548	100
**Level of education**				
Primary	133	26.9	137	25.0
Secondary	187	37.8	284	51.8
Tertiary college	152	30.8	107	19.5
University	14	2.8	1	0.2
None	8	1.7	19	3.5
**Total**	494	100	548	100

### Hospital length of stay by demographic and injury characteristics

The length of stay (LOS) in hospitals ranged from 1 to 235 days with a mean of 19.8 days± SD of 8.23, median of 9 days; with the majority 285(57.7%) being admitted for two weeks and below. Forty two (8.5%) patients died in the hospital. The association between the study variables and LOS is presented in Table 2. Among motorcycle injuries who recorded longer length of stay of 2 weeks or more, greater proportions were males 158 (75.6%), Motorcycle rider 98(46.9%), Head &neck injuries 96 (45.9%) patients demanding surgical interventions 193 (92.3%). A greater proportion of motorcycle injury patient had no health insurance. Of the patients who stayed in the hospitals for ≥2 weeks a greater proportion had no health insurance 109 (52.2%) vs 100(47.8%). Gender, anatomic injury site, road user category, ISS (p < 0.001), GCS, (p < 0.001), insurance status, (p < 0.001), helmet use, (p < 0.001), and surgery (p < 0.001) were significantly associated with LOS (Table 3).

**Table 3:** Hospital length of stay by demographic and injury characteristics (n=494)

	Length of stay		Mean length of stay	ANOVA (F test)	P value
	< 2 weeks (n=285)	≥2 weeks (n=209)			
**Gender**					
Male	225(78.9	158(75.6)	23.1	30.2	0.001
Female	60(21.1)	51(24.44)	14.2		
Age in years					
0-10	7(2.6)	10(4.80)	13.6		
11-20	30(10.5)	25(12.0)	19.6	3.03	0.15
21-30	99(34.7)	71(34.0)	26.6		
31-40	56(19.6)	53(25.3)	20.2		
41-50	42(14.7)	22(10.5)	22.2		
Above 50	51(17.9)	28(13.4)	22.2		
Road user					
Motorcycle rider	103(36.1)	98(46.9)	18.6	45.3	0.001
Pillion passenger	97(34.1)	53(25.4)	23.6		
Pedestrian	81(28.4)	56(26.7)	22.2		
Bicyclist	4(1.4)	2(1.0)	6.8		
Anatomic Injury site					
Head &neck	185(64.9)	96(45.9)	15.5	1.04.9	0.001
Chest, abdomen, pelvis & spine	44(15.6)	33(15.7)	22.2		
Extremities	34(11.9)	44(21.0)	18.3		
Multiple injuries	22(7.7)	36(17.2)	24.7		
**Injury severity(ISS)**					
<16	116(40.7)	82(39.2)	4.4	305.8	0.001
>16	169(59.3	127(60.8)	28.5		
**Glasgow Coma**					
13-15	63(22.1)	53(25.4)	5.3	258.8	0.001
9-12	50(17.5)	28(13.4)	15.6		
3-8	172(60.4)	128(61.2)	25.6		
Health insurance status					
Yes	129(45.3)	100(47.8)	15.4	2.8.1	0.001
No	156(54.7)	109(52.2)	25.3		
**Surgical interventions**					
Yes	278(97.5)	193(92.3)	28.3	1.158.3	0.001
No	7(2.5)	16(7.7)	5.3		
**Helmet use**					
Yes	34(11.9)	29(13.9)	4.5	.22.5	0.001
No	251(88.1)	180(86.1)	24.7		

### Mean and total medical costs based on socio-demographic and injury characteristics of motorcycle crash victims

As shown in Table 4, the distribution of patients patient charges varied considerably and was highly skewed, ranging from just 100 ($1) to 19, 134,877 Kshs (US$ 1US$ 103569.81) (USD 191348.77).. Overall, the mean cost per motorcycle patient visit was Kshs 17833.06 ($178.33.06) and varied by ED disposition: $97.00 for patients treated in ED and discharged; and Kshs. 41124.6 ($411.25) for patients admitted to the hospital. The mean medical cost was higher for male Kshs. 19594.17 ($195.94) than females Kshs. 14773.72($147.74)

**Table 4:** Mean and median medical cost based on socio-demographic and injury characteristics of motorcycle crash victims

Characteristic of the motorcycle injured cases	Mean Total cost (Kshs.)		P value
**Gender**			
Male	19594.17	15205077	0.001
Female	14773.72	3929800	
**Age of the motorcycle injury cases**			
0-10	18363.61	1395635	0.37
11-20	13208.91	1505816	
21-30	25652.01	8926900	
31-40	18776.83	4543995	
41-50	14575.23	1279270	
Above 50	17200.81	1479270	
**Anatomic body site of injury**			
Head and neck	21935.04	9826900	0.01
Chest, abdomen, pelvis, spine	12037.08	3450542	
Extremities	14140.47	1244361	
Multiple sites	27622.87	2983270	
**Helmet use**			
Yes	10749.42	2117632	0.01
No	20138.76	17017254	
**Glasgow Coma Scale**			
Mild(13-15)	9744.83	5116034	0.01
Moderate(9-12)	12503.04	2913209	
Severe(3-8)	41124.6	1110344	
**Injury severity (ISS)**			
<16	5089.41	2946772	0.001
>16	41292.1	16188105	
Health			
insurance			
Yes	42220	12198839	0.021
No	21173	6936038	

Mean motorcycle injury patient charges per type of injury ranged from Kshs. 14140.47 ($ 141.4047 for extremities to Kshs. 21935.04 ($ 219.35.04) for head injuries. Head injuries accounted for the greatest proportion (51.4%) of the total charges. Further analysis revealed that helmet non-use resulted into a total medical cost of Kshs. 17, 017, 254; which accounted for 88.9% of the total medical cost. The mean cost of motorcycle injuries was higher for patients who did not use helmets Kshs. 20138.76 ($201.39) compared with those who used helmets before the crash Kshs.10749.42 ($10749.42) respectively. Patients with ISS>16 also demonstrated higher mean medical cost of Kshs 41292.1($412.92) than patients who had less severe injuries ISS<16 and accounted for 84.5% of the total medical cost. More than half (57.5%) of the total medical costs were billed to government-sponsored health insurance programmes combined (NHIF, UHC county sponsored programme, with aean of Kshs. 4222($42.20) per visit.

### Predictors of hospital length of stay of motorcycle injuries

Analysis of the crude odds ratios for factors associated with hospital length of stay (Table 4) shows that injury severity, higher injury severity score of ISS > 16, and lower Glasgow Coma Scale; had 6 and 5 times increased influence on length of stay respectively, surgical intervention (OR =5.3; CI=0.9-10.27), helmet nonuse (OR =4.9; CI=3.3-10.29) motorcycle rider (OR =1.7; CI=0.467-3.771), also increases hospital length of stay. In terms of anatomic injury site, motorcycle injury patients who sustained injuries to the head were 5 times more likely to stay longer in the hospital than those who sustained injuries to the extremities.

After adjusting for the effects of all the variables, surgical intervention ((OR =6.5; CI=1.9-20.47), higher injury severity score (ISS>16), lower Glasgow Coma Scale; GCS=3-8 (OR =5.5; CI=2.24-16.82) were the main factors influencing hospital length of stay. In the final model, demographic factors, being male (OR = 1.7; CI= 1.95-3.38), age groups of 19-30 years (or =2.91; CI=1.48-7.92) and 31-40 years (OR =1.2; CI= 0.28-1.73) increased significantly the length of stay. In relation anatomic injury site; Head, face and neck (OR =4.6; CI= 2.74-6.15) and multiple injuries (OR =3.01; CI= 0.14-9.15) significantly increased the likelihood of staying longer in the hospital (Table 5).

**Table 5:** Predictors of hospital length of stay of motorcycle injuries

Gender	Crude Odds Ratio (95% Cl)	P value	Adjusted Odd Ratio (95% cl)	P value
Males	1.2(0.466-2.211)	0001	1.7(1.95-3.38)	0.001
Females	Ref		Ref	Ref
**Age**				
<18	0.4 (0.02-4.29)	0.35	0.3 (0.06-3.49)	0.28
19-30	2.6 (0.66-5.82)	0.030	2.9 (1.48-7.92)	0.01
31-40	0.9 (0.54-5.38)	0.106	1.2 (0.28-1.73)	0.05
41-50	0.2 (0.150-1.39)	0.45	0.16 (0.16-1.16)	0.42
above 50	Ref		Ref	Ref
**Type of road user**				
Motorcycle rider	1.7(0.467-6.171)	0.003	1.4(0.17-3.28)	0.03
Passengers	1.4(0.400-4.900)	0.56	1.0 (0.74-1.42)	0.9
Pedestrian	1.6(0.193-3.999)	0.455	1.7(0.14-4.36)	0.6
Bicyclist	ref	Ref	Ref	Ref
**Helmet use**				
Yes	ref		Ref	ref
No	4.9(3.3-10.29)	<0.001	5.2(0.36-12.70)	0.001
**Anatomic injury site**				
Head, face and neck	5.4(0.567-11.17)	0.05	4.6(2.74-6.15)	0.001
Chest/thorax/abdomen	2.5(0.26-7.16)	0.08	2.6(0.23-8.35)	0.18
Multiple injuries	4.2(1.70-10.57)	0.001	3.01(0.14-9.15)	0.05
Extremities	ref	ref	ref	ref
**Injury severity(ISS)**				
>16	6.0(2.06-11.047)	0.001	6.2 (1.04-12.65)	0.001
<16	Ref	Ref	Ref	Ref
**Glasgow Coma Scale**				
3-8	5.3(2.20-10.73)	0.01	5.5(2.24-16.82)	0.001
9-12	0.8(0.07-1.708)		1.05(0.37-4.36)	0.041
13-15	Ref	Ref	Ref	
**Surgical intervention**				
Yes	5.3(0.9-10.27)	0.001	6.5(1.9-20.47)	
No	Ref	Ref	Ref	
**Pre hospital care obtained**				
No	3.10(1.18-8.58)	0.004	2.2(1.25-6.74)	0.001
Yes	Ref		Ref	Ref
N/A				
**Health insurance**				
Yes	0.7(0.39-1.38)	0.612	0.6(0.26-1.67)	0.07
No	Ref		Ref	Ref
Hospital type				
Public	-0.3 (-0.43 -0.50)	0.01	0.4(-0.39-1.38)	0.01
Private	Ref	Ref	Ref	Ref

### Predictors of hospital medical charges of motorcycle injuries

Analysis of the crude odds ratios for factors associated with medical costs (Table 6) shows that surgical intervention increased medical charges 18.3 times (OR = 18.3; CI=3.96-90.59),

**Table 6:** Multivariable analysis of predictors of hospital medical charges of motorcycle injuries

Gender	Crude Odds Ratio (95% Cl)	P value	Adjusted Odd Ratio (95% cl)	P value
Males	0.7(0.498-0.998)	0.049	1.2(0.45-2.08)	0.04
Females	Ref		Ref	Ref
**Age**				
<18	0.4(0.02-4.29)	0.3	0.3(0.06-3.49)	0.28
19-30	2.5(0.47-5.32)	0.03	2.7(1.08-4.57)	0.01
31-40	0.8(0.56-7.38)	0.11	1.0(0.28-1.03)	0.35
41-50	0.2(0.150-1.39)	0.45	0.130(0.06-0.09)	0.42
above 50	Ref		Ref	Ref
**Type of road user**				
Motorcycle rider	0.5(0.067-1.141)	0.01	1.2(0.67-1.68)	0.001
Passengers	0.6(0.040-1.340)	0.321	1.0 (0.04-0.99)	0.96
Pedestrian	1.3(0.243-2.999)	0.05	1.1(0.14-4.36)	0.6
Bicyclist	ref	Ref	Ref	Ref
**Helmet use**				
Yes	ref		Ref	ref
No	5.9 (3.3-18.29)	<0.001	5.2(2.36-11.70)	<0.001
**Anatomic injury site**				
Head, face and neck	2.9(1.87-4.74)	0.025	0.6(0.04-1.15)	<0.301
Chest/thorax/abdomen	1.9(0.84-4.329)	0.05	0.7(0.03-1.55)	0.28
Multiple injuries	1.9(1.079-4.312)	0.01	3.01(0.14-9.15)	0.05
Extremities	ref	ref	ref	ref
**Injury severity(ISS)**				
>16	6.0(-0.06-0.047)	0.001	6.2 (1.04-12.65)	0.001
<16	Ref	Ref	Ref	Ref
**Glasgow Coma Scale**				
1-8	0.6(0.149-2.528)	0.51	2.5 (1.24-5.82)	<0.001
9-12	1.4 (0.357-6.279)	0.000	0.7 (0.07-1.28)	0.231
13-15	Ref	Ref	Ref	
**Surgical intervention**				
Yes	18(3.96-90.59)	0.001	18.5(0.9-92.49)	<0.001
No	Ref	Ref	Ref	
**Pre hospital care obtained**				
No	4.10(1.25-18.67)	0.004	4.2(1.37-17.62)	0.001
Yes	Ref		Ref	Ref
N/A				
**Medical insuranc**e				
Yes	0.8(0.57-1.048)	0.06	0.6 (0.26-1.67)	
No	Ref		Ref	Ref
**Hospital fatality**				
Yes	0.7(0.079-8.012)	0.074	1.3(1.079-2.022)	0.05
No	Ref		Ref	Ref
**Length of stay**				
> 2 weeks	17.7(1.23-90.72)	0.016	17.9(2.31-91.02)	
< 2 weeks	Ref			
Hospital type				
Public	0.2(-0.13 -0.10)	0.01	0.2(-0.19-0.18)	0.01
Private	Ref	Ref	Ref	Ref

Furthermore, hospital medical charges had a direct relationship with severity, motorcycle injury patients who sustained injuries with higher injury severity score ISS >16 (OR=6.0; 2.06-11.047) and lower Glasgow Coma Scale GCS 3-8 OR=1.4 (Cl 95%; 0.357-6.279) had increased probability to pay higher medical charges. In addition, all motorcycle crash injured areas were compared to injuries to extremities; the results indicated that the patients with head and neck injuries and Chest/thorax/and abdomen were 2.9 times and 1.9 respectively more likely to pay higher charges compared to extremities injuries. Moreover, the motorcycle injury patients who did not wear helmet were 5.9 times more likely to pay higher hospital charges (p < 0.001) than those who wore helmets.

After adjusting for the influence of all the factors in multivariable regression analysis, surgical intervention, longer length of stay > 2 weeks, higher injury severity score (ISS>16), helmet non-use, injuries to extremities, remained as the most important factors influencing hospital length of stay (Table 6).

## Discussion

Motorcycle injuries is an important cause of hospital admission and bed occupancy in Kenya. To our knowledge, this is the first study in the Kisumu City and in Kenya and among few studies in WHO-Africa region to explore factors influencing the length of stay and medical cost of motorcycle injuries. Hospitalization, due to motorcycle injuries, can be economically exigent in case of prolonged hospital stay. In this study, the average length of stay was approximately 19.8 days. This compares favorably with 18.3 and 19.2 days reported from Tanzania^47^ and Brazil^48^. Hospitals studies conducted in Kenya have reported mean LOS ranging from 14 to 24.3 days for motorcycle injury patients^27^,^39^, which falls within the average Length of stay (LOS) for motorcycle injuries reported in this study. On the other hand, the mean LOS was higher than reported in Iran^39^, and in USA^19^,^20^ these differences may be due to variations in injury patterns, case severity, trauma care organization, availability of technology, operating theater time and staff, and bed capacity/occupancy. This study found that the mean patient cost of hospitalized motorcycle injury cases was Kshs. 17833.06 (USD 178.33) which was lower compared to estimates reported from USA (19 20); Spain^49^; New Zealand^50^ and Iran^51^. This variation in hospital charges, could be due by the subsidized health care system and also the general low service costs since the study was done during the implementation of Universal Health UHC) pilot. However the rising number of motorcycle related injuries and fatalities in Kenya, exert huge economic burden to individuals, families, society and Government. The study further showed that hospital length of stay and medical cost of motorcycle injuries varied by surgical procedures, injury severity (higher ISS and lower GCS), age, gender, injury characteristics, health outcome of patients, and type of road users, helmet use.

In agreement with Kashkooe at al^52^, this study revealed that motorcycle injury patients who were under surgical procedures were 5.3 and 18.5 times more likely to stay longer in hospital and pay higher medical charges respectively, with victims often requiring more than one surgical intervention. On the contrary, to a study by Schuurmans et al^53^ that indicated that surgical procedures on rib fractures and injuries are effective in reducing LOS. This discrepancies could be attributed to some surgical complications, which could require more hospital days ranging from 2-3 weeks.

The hospital of care (being Tie III hospitals) was a predictor of both length of stay and medical cost. This might have been due to the fact that more serious cases ended up at these hospitals. Likewise, motorcycle injuries with higher ISS and lower GCS scores had increased length of stay and medical cost, this was in line with previous studies^20^,^52^,^54^,^55^, and 56; which showed that increased injury severity resulted to increased LOS and medical cost in trauma care settings. Such injuries are more serious cases which require greater interventions. Patients with low ISS have less forms of injuries, and generally, recover fast, while those with greater scores are more likely to have a longer period of recuperation. This study shows that in the absence of Injury Severity Score,^41^, LOS can be used as a proxy in epidemiological studies as a metric of hospital utilization and characterize patients with severe injuries. However, it is important to establish and maintain minimum admission criteria, standardized inpatient management practices and patient discharge procedures across all hospitals whether private or public in order to improve the validity of using the mean length of stay as an indicator of both injury severity and hospital utilization.

In agreement with previous study^18^,^57^,^58^, motorcycle injury victims in the current study were predominantly male (male–female ratio 3.9), mainly in productive age. Most hospitalized motorcycle injuries (77.5%) occurred to men, the costs and length of stay of whom were 1.2 times and 1.7 times respectively greater than those of females. This is because men tend to spend most of time out of home than females and are more likely to be exposed to road traffic crashes.

The study revealed that length of stay was a significant factor in medical charges. Hospital length of stay and ICU admission has been reported in previous studies to be associated with acute treatment costs^49^,^59^. For example, reduced hospital LOS has been correlated with reduced costs^15^, while prolonged LOS is related to increased demand for resources both material and manpower^14^. Therefore, LOS is a useful indicator for costs, and hospital resource utilization^16^.

In consistent with other studies^11^,^60^, this study found that considrable increase in the length of stay and medical cost is attributed to head injuries, multiple injuries and injuries to the extremities. Brain injury and multiple injuries due to motorcycle trauma have been associated with high demand for specialized medical care and procedures, longer hospital length of stay which result in higher treatment costs. Multiple injuries often require, multiple radiological and laboratory investigations, multiple surgical interventions, and prolonged ICU and hospital stay.

A striking feature in this study was the low coverage of health insurances. The study found that 52.2% of the patients were not covered by any type of health insurance of which 41.1% stayed in the hospital for more than 2 weeks. The findings showed that un- insured motorcycle injury patients stayed longer in hospital compared to the insured. This could be because some patients were detained for inability to promptly settle hospital bills. Insurance status has been indicated to be an important factor influencing utilization of health care services and lack of it would pose barrier to health care.

The findings indicated that total medical cost and Length of stay varied with road users' category. Compared with other road users, injuries to the motorcycle rider, pillion passengers and pedestrian was associated with higher hospital charges and longer hospital stay. Longer LOS for motorcycle rider and pedestrian has also been reported in previous studies^46^,^61^. Higher injury rates and severity levels among motorcycle riders are related to several factors, such as driving inexperience, risk-taking behavior, and greater risk exposure (wearing safety belts less, driving while intoxicated^51^. Longer length of stay among pedestrian has also been reported in^49^, ^50^, ^51^ studies. Pedestrians and motorcycle riders frequently sustain injuries to the head, spine and lower extremity demanding expensive diagnostic and therapeutic procedures.

Previous studies have reported that motorcycle crash injuries resulting in death are associated with higher charges and longer length of stay^21^, ^49^, ^52^, ^62^, and 63. Motorcycle injury patients who died in the hospital during this study incurred significantly higher charges but had shorter LOSs when compared with patients who survived recuperating with their injuries. This could be attributed to use of costly intensive-care diagnostic and management procedures, resulting in higher charges. However, other studies have been Kisat et al.,^60^ revealed that injured adult trauma patients who did not die within the first few days and had longer LOS demonstrated a higher ability to survive and demanded medical care for extended period of time.

The findings revealed that use of helmet among the motorcycle injury patients was very low and had significant effect on length of stay and medical costs, and are in agreement with previous studies which reported that not wearing a safety helmet is associated with higher hospital charges and longer LOS^64^,^65^. Mean length of stay of 24.7 days and 8.6 days was observed among un-helmeted and helmeted motorcycle injury cases respectively which was higher than 12.6 days and 11.8 days for un-helmeted motorcycle injury cases and 9.9 days and 5.8 days for those who wore helmets reported in other studies^66^,^67^. These findings indicate that wearing a helmet prevent severe head injury and reduce injury severity among motorcycle riders and subsequent hospitalization. The effectiveness of helmet laws in improving the use of helmets by motorcycle riders and passengers and reducing head injuries has been reported in other studies^68^,^69^. Traffic amendment bill in Kenya, passed in 2009, incorporated the mandatory use of helmets for all motorcycle riders and their pillion passengers and increased the penalties in the Traffic Act in 2012. But the wearing rate remains low. There is need for strict enforcement and increased publicity of the law to improve compliance. Increased enforcement has been shown to be one of the most effective methods to increase usage of safety equipment^69^. The strength of this study was that all motorcycle injuries patients presenting in three Tier III hospitals were enlisted in the study. Enlisting all actual cases has been documented to provide a much more representative data on the burden of motorcycle crashes. The hospitals receive and manage large number of trauma patients, on a 24 hour basis.

This was done purposely so as to focus on motorcycle patients with injuries severe enough to require hospital services: moreover, emergency departments provide the best opportunity for capturing data on a wide range of non-fatal injuries. Cost estimation was done on data captured from actual costs generated from each services rendered therefore curtailing the effected of recall bias. These results should be viewed considering few limitations. First, the study focused only on motorcycle injury patients who sought and obtained care in three major referral hospitals, and nothing is known about those seeking care elsewhere. The motorcycle injury cases are likely to be under-estimates and therefore the results may not be generalized to the wider national population. Nevertheless useful data on injured patients choosing to seek care at the hospitals could be obtained and the consequent burden on the hospitals quantified. However, these hospitals were chosen deliberately in order to focus on motorcycle casualties with injuries severe enough to demand hospital services in emergency departments, since emergency departments provide the best opportunity for capturing data on a wide range of non-fatal injuries. Helmet use was not objectively verified since they were self-reported by the injury patients.

## Conclusion

Motor cycle crash–related injury in the Kenya results in high morbidity, hospitalization, mortality, and economic costs. Surgical intervention, injury severity, non-helmet use, being motorcycle rider are the major predictors of length of stay and medical costs. Data on admission, length of stay and cost should be taken into account to ensure relevance, adequacy, and responsiveness of trauma delivery services. It is anticipated that this data can assist in planning for patients care, designing necessary measures for controlling crashes that increase spending on preventable conditions thus reducing hospital costs of motorcycle injury victims.

### Dissemination plan

Results of the study will be presented to different stakeholders including clinical teams at the hospitals where data were collected in Kisumy City, Kenya where it was conducted during their clinical grand rounds. We will also present at emergency medicine conferences in Africa and internationally

## Data Availability

Data is contained within this article but can be availed on request from the investigator
